# Effect of acidified turmeric and/or black pepper on growth performance and meat quality of broiler chickens

**DOI:** 10.1080/23144599.2020.1830691

**Published:** 2020-10-13

**Authors:** Sugiharto Sugiharto, Anugrah Robby Pratama, Turrini Yudiarti, Hanny Indrat Wahyuni, Endang Widiastuti, Tri Agus Sartono

**Affiliations:** Department of Animal Science, Faculty of Animal and Agricultural Sciences, Diponegoro University, Semarang, Indonesia

**Keywords:** Acidification, *averrhoa bilimbi* Linn. fruit filtrate, broiler, growth, herbs, meat quality

## Abstract

The study investigated the effect of acidified turmeric, black pepper or its combination on growth and meat quality of broilers. The *Averrhoa bilimbi* Linn. fruit filtrate was used to acidify the herbs. A number of 392 day-old Lohmann broiler chicks were randomly distributed to four groups, including CONT (control diet), TRMC (diet supplemented with 1% acidified turmeric), BLPR (1% acidified black pepper) and TRPR (1% acidified turmeric and 1% acidified black pepper). Body weight, feed intake and feed conversion ratio (FCR) were weekly recorded. Internal organ weight and carcase traits were determined at day 35. The CONT and TRMC showed greater (p < 0.05) weight gain than BLPR and TRPR. The FCR was lower (p < 0.05) in TRMC than in BLPR and TRPR, but did not differ from CONT. The gizzard was greater (p < 0.05) in BLPR than that in CONT and TRMC. The BLPR had smaller (p < 0.05) pancreas than other chickens. Abdominal fat was lower (p < 0.05) in TRMC, BLPR and TRPR than that in CONT, of which BLPR was the lowest. Drumstick was greater (p < 0.05) in BLPR than in CONT. CONT had lighter and less yellow (p < 0.05) breast meats than other broilers. In thigh meats, the lightness (L*) values were higher (p < 0.05) in CONT than in TRMC and BLPR. The yellowness (b*) were lower (p < 0.05) in CONT than in TRPR meats. In conclusion, acidified turmeric reduced abdominal fat deposition and improved meat quality of broilers.

## 1. Introduction

The use of herbs or phytogenic materials as feed additive has commonly been practiced to improve the growth performance of broiler chickens. Turmeric is one of the most common herbs that has been used as feed additive for broilers [1]. However, the growth-promoting effect of turmeric seems to be inconsistent, as many investigators underlined the failure of turmeric in improving the growth performance of broiler chickens [2–4]. Indeed, the poor bioavailability, solubility and stability of curcumin can hinder the phytobiotic activities of the herb *in vivo* [5].

Strategies have been developed to enhance the bioavailability of curcumin, one of which is by combining of curcumin with other herb such as black pepper [5]. As a note, black pepper contains piperine that is known as bioavailability enhancer [6]. However, different from the expected impact, the combination of turmeric and black pepper may not always improve broiler chicken performance as Akbarian et al. [7] and Abou-Elkhair et al. [8] did not find any effect of the combination of turmeric and black pepper on growth performance of broilers. Studies showed that the nutritional and functional properties of plant-derived products may be improved through acidification. Sarr and Tsai [9] formerly reported that acidification resulted in higher nutritional factors and antioxidant capacities of tomato juice, while Bayliak et al. [10] found the increased antioxidant capacities in medicinal herbs with acidic treatment. In accordance, the solubility and stability of curcumin increased in the acid solution [11,12]. Recently, there has been a trend towards minimizing the use of chemical and rather shifting to natural constituents as dietary additives for broilers [13]. Besides turmeric and black pepper, there is still a wide range of potential medicinal plants in Indonesia that can be exploited as dietary additives for broilers, one of which is *Averrhoa bilimbi* (*Averrhoa bilimbi* Linn.) fruit. The juice of *A. bilimbi* fruit is sour and extremely acidic [14], and can therefore be used to acidify the turmeric and black pepper.

Administration of herbs has been documented to improve carcase and meat quality of broilers. Kanani et al. [15] reported that feeding turmeric powder improved meat quality (i.e., decreased lightness values, increased pH and dry matter content of meats), while Ndelekwute et al. [16] showed that black pepper powder increased carcase percentage, breast and thigh, and also decreased abdominal fat of broilers. Yet, inconsistent findings were reported. Hidayat et al. [17] did not see any effect of turmeric extract on carcase and meat quality of broilers. Also, Singh et al. [18] reported no influence of black pepper powder on carcase characteristics and abdominal fat content of broilers. In the current study, dietary administration of acidified herbs was expected to consistently improve the carcase and meat characteristics of broilers, in addition to the growth rate of broilers. To best of our knowledge, no other study reporting the use of acidified herbs on broilers so far. The aim of the present study was therefore to investigate the effect of acidified turmeric, black pepper or its combination on growth performance and meat quality of broiler chickens.

## 2. Materials and methods

### 2.1. Production of acidified herbs

*A. bilimbi* fruit filtrate was initially prepared before the production of acidified herbs. The fruit filtrate was produced from ripe *A. bilimbi* fruit harvested from the gardens around the university. After being washed thoroughly (using running water) and drained, the fruit was blended using a medium-speed electronic blender. Water was not added during juicing. The fruit juice was then filtered using cheesecloth to obtain a fruit filtrate.

Turmeric and black pepper powders were bought from the local market in Semarang, Central Java, Indonesia. The acidified herbs were produced by mixing turmeric or black pepper powders with *A. bilimbi* fruit filtrate as prepared previously (1:3; g:mL). The mixture was placed in an anaerobic jar and anaerobically incubated for 4 days at room temperature (±25ºC). The mixture was sun-dried and then stored at refrigerator (±4ºC) until use. Samples of acidified herbs were also obtained for pH, total acids and antioxidative properties.

The pH of herbs was measured using a digital pH metre (Eco Test pH 1, Thermo Fisher Scientific Inc.). Total acidity of the herbs was determined based on the titration procedure as described by Apriyantono et al. [19]. The total acidity was determined by neutralizing the acid contained in the herb samples using 0.1 M NaOH standardized with potassium hydrogen phthalate. The colour change of phenolphthalein indicated the endpoint of titration. To measure the antioxidant capacity, the herbs was subjected to the 2,2-diphenylpicrylhydrazyl (DPPH) free radical scavenging assay [20]. The absorbance was determined at 515 nm. Ascorbic acid (Sigma-Aldrich, St. Louis, MO, USA) that is a standard antioxidant was employed as a reference. The phenolic content of herbs was assessed according to Folin-Ciocalteu method [21]. The herb (0.5 mL) was mixed with 8 mL of distilled water, 0.5 mL of Folin-Ciocalteu reagent (Merck KGaA, Darmstadt, Germany) and 1 mL of sodium carbonate (Na_2_CO_3_, Merck KGaA). The mixture was incubated at room temperature (±25ºC) for 30 min. The absorbance was then determined at 765 nm using a spectrophotometer. Gallic acid was used to make standard curve.

### 2.2. In vivo experiment

The study was approved by the Animal Ethics Committee of the Faculty of Animal and Agricultural Sciences, Diponegoro University and conducted in compliance with the standard rearing protocols of livestock (the law of the Republic of Indonesia number 18, 2009). The experiment was designed based on a completely randomized design. A total of 392 day-old Lohmann broiler chicks (mixed sex; average body weight of 38.98 ± 1.14 g; means ± standard deviations) were randomly distributed to one of four treatment groups, each consisting of 7 replications with 14 chicks in each. The treatment groups included CONT (control diet, without any additive), TRMC (diet supplemented with 1% acidified turmeric), BLPR (diet supplemented with 1% acidified black pepper) and TRPR (diet supplemented with 1% acidified turmeric and 1% acidified black pepper). Throughout the rearing period, the chicks were grown in an opened-sided broiler chicken house with rice husk as bedding material. The broiler chickens were raised in 1.10 × 1.10 m pen equipped with manual feeder and drinker. Continuous lighting programme was applied during the whole experimental period. From day 1–7, the chicks were offered with commercial pre-starter diet (containing 23% crude protein, 5% crude fibre, 5% crude fat and 7% ash). The chicks were provided with formulated starter and finisher diets (Table 2) from days 8 to 21 and days 22 to 35, respectively. The feed formulations were carried to meet the Indonesian National Standards for broiler feed [22]. The acidified herbs were added (“on top”) to feeds (commercial or formulated rations) and offered for the entire experiment (days 1–35). In this study, we did not calculate the nutrient values of the additives in our feed formulations. The chicks were vaccinated using Newcastle disease vaccine through eye drops (day 4) and drinking water (day 18). Vaccination using infectious bursal disease vaccine was also conducted on day 12 through drinking water.

**Table 1.** pH, total acids and antioxidative properties of acidified herbs^1^

Items	Raw herbs	Acidified herbs
Turmeric powder		
pH	5.90 ± 0.00	3.70 ± 0.00
Total acidity (%)	2.32 ± 0.04	6.50 ± 0.12
Antioxidant activity (IC_50_, ppm)^a^	67.8 ± 4.35	89.8 ± 12.0
Total phenolics (%)	3.48 ± 0.01	3.39 ± 0.05
Black pepper powder		
pH	5.80 ± 0.00	3.00 ± 0.00
Total acidity (%)	1.16 ± 0.03	3.49 ± 0.05
Antioxidant activity (IC_50_, ppm)^a^	1,298 ± 41.5	1,118 ± 2.77
Total phenolics (%)	0.36 ± 0.00	0.47 ± 0.04

Key: ^1^Data are presented as means ± standard deviations and not statistically analysed. Analysis was conducted on two samples

^a^IC_50_ is considered as the potent concentration at which the 2,2-diphenylpicrylhydrazyl (DPPH) radicals were neutralize by 50%. A greater of radical neutralizing activity of DPPH is attributed to a lower IC_50_ value.

**Table 2.** Ingredients and chemical compositions of diets

Items (%, unless otherwise noted)	Starter (8–21)	Finisher (22–35)
Yellow maize	55.9	62.5
Soybean meal	37.1	29.5
Palm oil	2.22	3.22
DL-methionine, 990 g	0.19	0.19
Bentonite	1.00	1.00
Limestone	1.34	1.34
Monocalcium phosphate	1.51	1.51
^1^Premix	0.27	0.27
Chlorine chlorite	0.07	0.07
Salt	0.40	0.40
Calculated chemical compositions:		
^2^ME (kcal/kg)	2,900	3,040
Crude protein	21.0	18.0
Crude fibre	5.50	5.50
Ca	1.30	1.30
P	0.60	0.60
Analysed chemical compositions:		
^2^ME (kcal/kg)	3,144	3,219
Dry matter	90.1	89.2
Crude protein	19.0	16.9
Crude fat	3.30	3.44
Crude fibre	8.69	9.40
Crude ash	9.77	7.10

Key: ^1^Premix consisted (per kg of feed) of vitamin A 7,750 IU, vitamin D_3_ 1,550 IU, vitamin E 1.88 mg, vitamin B_1_ 1.25 mg, vitamin B_2_ 3.13 mg, vitamin B_6_ 1.88 mg, vitamin B_12_ 0.01 mg, vitamin C 25 mg, folic acid 1.50 mg, Ca-d-pantothenate 7.5 mg, niacin 1.88 mg, biotin 0.13 mg, BHT 25 mg, Co 0.20 mg, Cu 4.35 mg, Fe 54 mg, I 0.45 mg, Mn 130 mg, Zn 86.5 mg, Se 0.25 mg, L-lysine 80 mg, Choline chloride 500 mg, DL-methionine 900 mg, CaCO_3_ 641.5 mg, DCP 1500 mg

^2^ME (metabolizable energy) was determined based on formula [46] as follow: 40.81 {0.87 [crude protein + 2.25 crude fat + nitrogen‐free extract] + 2.5}

The body weight of chicks, feed intake and feed conversion ratio (FCR) were recorded on weekly basis throughout the experiment. On day 35, 2 male chickens representing the average body weight of each pen (14 chicks per treatment group) were taken and slaughtered. The male broiler chickens were selected to minimize the bias particularly on meat quality due to gender variations. Two broiler chickens from each pen were used as the samples for measurement to increase the sample size and thus decrease the margin of error. After de-feathering and dissecting, the internal organs of broiler chickens were removed. The internal organs were weighed and internal organs relative weight was calculated as described by Sugiharto et al. [23]. The carcase and commercial cuts of each broiler chicken were determined thereafter. Samples of breast and thigh meats were collected for the determination of meat colour.

The colour of meats was determined using a digital colour metre in Mac OS X (set to CIE Lab) as previously conducted by Sugiharto et al. [24]. The meat colour was presented as L* (lightness), a* (redness) and b* (yellowness) values.

Data were treated based on analysis of variance (ANOVA, SPSS 16.0 version [25]). Duncan multi-range test was performed when the significant influence (p < 0.05) of dietary treatments was observed.

## 3. Results

### 3.1. Productive performance of broilers

Data on the performances of broilers are presented in Table 3. At day 1–21, CONT and TRMC had higher (p < 0.05) weight gain and lower (p < 0.05) FCR than BLPR and TRPR chicks. At days 22–35, CONT and TRMC had higher (p < 0.05) weight gain than TRPR chicks, but did not differ from BLPR chicks. At days 22–35, FCR was lower (p < 0.05) in TRMC than in TRPR, but was not different (p > 0.05) from CONT and BLPR broiler chickens. For the entire period (days 1–35), CONT and TRMC showed greater (p < 0.05) weight gain than BLPR and TRPR broilers. The FCR was lower (p < 0.05) in TRMC than in BLPR and TRPR, but did not differ from CONT broiler chickens. Cumulative feed intake was not different (p > 0.05) among the dietary treatment groups throughout the experimental period. Mortality was absent during the study.

**Table 3.** Performances of broilers

Items	Dietary treatments				SEM	p value
	CONT	TRMC	BLPR	TRPR		
Days 1–21						
Weight gain (g)	633^a^	639^a^	566^b^	573^b^	8.18	<0.01
Cumulative FI (g)	963	942	975	938	8.30	0.36
FCR	1.53 ^c^	1.48 ^c^	1.73^a^	1.64^b^	0.02	<0.01
Days 22–35						
Weight gain (g)	897^a^	883^a^	838^ab^	775^b^	17.0	0.04
Cumulative FI (g)	2,111	1,929	2,061	2,041	25.9	0.08
FCR	2.37^ab^	2.19^b^	2.50^ab^	2.65^a^	0.06	0.04
Days 1–35						
Weight gain (g)	1,530^a^	1,522^a^	1,404^b^	1,348^b^	21.6	<0.01
Cumulative FI (g)	3,075	2,870	3,036	2,980	29.9	0.08
FCR	2.02^ab^	1.89^b^	2.18^a^	2.21^a^	0.04	<0.01
Mortality (%)	0	0	0	0	0	

Key: ^a,b,c^Values with divergent letters within the same row are obviously different (p < 0.05)

CONT: control diet, TRMC: diet supplemented with 1% acidified turmeric, BLPR: diet supplemented with 1% acidified black pepper, TRPR: diet supplemented with 1% acidified turmeric + 1% acidified black pepper, BW: body weight, FI: feed intake, FCR: feed conversion ratio, SEM: standard error of the means

### 3.2. Internal organ weight

The relative weight of internal organs of broiler chickens are listed in Table 4. The relative weight of gizzard was greater (p < 0.05) in BLPR than that in CONT and TRMC, but did not differ (p > 0.05) from that in TRPR broiler chickens. The BLPR had lower (p < 0.05) pancreas weight than other broiler chickens. Abdominal fat content was greatest (p < 0.05) in CONT than in other birds. The relative weights of heart, liver, proventriculus, small intestinal segments, caeca and immune organs of broilers did not differ (p > 0.05) among the treatment diets.

**Table 4.** Relative weight of internal organs of broilers

Items (% live BW)	Dietary treatments				SEM	p value
	CONT	TRMC	BLPR	TRPR		
Heart	0.47	0.46	0.45	0.46	0.01	0.86
Liver	2.16	2.13	2.11	2.25	0.05	0.72
Proventriculus	0.51	0.51	0.48	0.49	0.01	0.69
Gizzard	1.32^b^	1.43^b^	1.63^a^	1.49^ab^	0.04	0.01
Pancreas	0.25^a^	0.24^a^	0.19^b^	0.24^a^	0.01	0.03
Abdominal fat	1.48^a^	1.22^b^	0.94 ^c^	1.20^b^	0.04	<0.01
Duodenum	0.49	0.47	0.53	0.46	0.02	0.26
Jejunum	0.98	0.96	1.04	0.97	0.02	0.43
Ileum	0.43	0.44	0.52	0.45	0.02	0.41
Caeca	0.78	0.75	0.82	0.72	0.02	0.42
Spleen	0.11	0.15	0.10	0.08	0.01	0.16
Thymus	0.24	0.20	0.18	0.19	0.01	0.40
Bursa of Fabricius	0.13	0.14	0.16	0.14	0.01	0.58

Key: ^a,b,c^Values with divergent letters within the same row are obviously different (p < 0.05)

CONT: control diet, TRMC: diet supplemented with 1% acidified turmeric, BLPR: diet supplemented with 1% acidified black pepper, TRPR: diet supplemented with 1% acidified turmeric + 1% acidified black pepper, BW: body weight, SEM: standard error of the means

### 3.3. Carcase characteristics of broilers

The proportion of drumstick was higher (p < 0.05) in BLPR than in CONT, but was not different (p > 0.05) from TRMC and TRPR chicks (Table 5). The eviscerated carcase and the proportions of breast, wings, thigh and back were not different (p > 0.05) across the dietary treatment groups.

**Table 5.** Carcase characteristics of broilers

Items	Dietary treatments				SEM	p value
	CONT	TRMC	BLPR	TRPR		
Eviscerated carcase (% live BW)	68.0	66.9	66.3	67.8	0.36	0.28
	% eviscerated carcase					
Breast	35.5	35.8	34.6	35.0	0.28	0.41
Wings	11.0	11.0	11.5	11.1	0.12	0.53
Thigh	17.0	17.3	17.5	17.6	0.19	0.68
Drumstick	13.8^b^	14.3^ab^	15.0^a^	14.6^ab^	0.15	0.02
Back	22.7	21.6	21.5	21.7	0.25	0.16

Note: ^a,b^Values with divergent letters within the same row are obviously different (p < 0.05)

CONT: control diet, TRMC: diet supplemented with 1% acidified turmeric, BLPR: diet supplemented with 1% acidified black pepper, TRPR: diet supplemented with 1% acidified turmeric + 1% acidified black pepper, BW: body weight, SEM: standard error of the means

### 3.4. Meat colour of broilers

The data on meat colour of broiler chicken meats are shown in Table 6. The L* values of breast meats were higher (p < 0.05) while the b* values were lower (p < 0.05) in CONT than in other treated broiler chickens. In thigh meats, the L* values also higher (p < 0.05) in CONT than in TRMC and BLPR, but did not differ (p > 0.05) from that in TRPR meats. The b* values were lower (p < 0.05) in CONT than in TRPR meats, but were not significantly different from the TRMC and BLPR meats. The a* values of breast and thigh meats were not different (p > 0.05) among the dietary treatment groups.

**Table 6.** Meat colour of broilers

Items	Dietary treatments				SEM	p value
	CONT	TRMC	BLPR	TRPR		
Breast meat						
L*	55.1^a^	50.7^b^	51.8^b^	50.3^b^	0.34	<0.01
a*	0.32	0.38	1.56	0.82	0.21	0.15
b*	6.96^b^	9.38^a^	9.84^a^	10.1^a^	2.19	<0.01
Thigh meat						
L*	52.2^a^	48.9^b^	48.8^b^	50.7^ab^	0.36	<0.01
a*	3.36	3.49	4.41	4.03	0.29	0.55
b*	6.48^b^	7.15^ab^	7.63^ab^	8.57^a^	0.25	0.03

Key: ^a,b^Values with divergent letters within the same row are obviously different (p < 0.05)

CONT: control diet, TRMC: diet supplemented with 1% acidified turmeric, BLPR: diet supplemented with 1% acidified black pepper, TRPR: diet supplemented with 1% acidified turmeric + 1% acidified black pepper, L*: lightness values, a*: redness values, b*: yellowness values, SEM: standard error of the means

## 4. Discussion

Acidification using *A. bilimbi* fruit filtrate resulted in decreased pH values and increased total acids in turmeric and black pepper powders. This could be understood as the acid characteristic of *A. bilimbi* fruit filtrate (pH value ranges from 0.9 to 1.5 [26]), which could therefore acidify the herbs. Moreover, the high natural organic acid (particularly citric acid [26]) content in *A. bilimbi* fruit filtrate may increase the total acids content in the *A. bilimbi* fruit filtrate-acidified herbs. However, acidification did not have substantial effect on the antioxidant activity and total phenolics of turmeric and black pepper powders in the present study. Data on the antioxidant activities and total phenolics of the herbs are presented in Table 1. This finding was different from Bayliak et al. [10] reporting the increased antioxidant capacities of medicinal herbs in acidic pH solvent. The difference in pH values of media (to dissolve the herbs) seemed to be responsible for the discrepancy results above. Indeed, Sun et al. [27] reported that total phenolic compounds and antioxidant activity of sweet potato leaf polyphenols increased in neutral and weak acid solvent systems, and the optimum pH values for increasing the total phenolics and antioxidant activity of the herbs was 5.0–7.0. The optimum pH values (for increasing the antioxidant capacity of herbs) were by far higher than the pH values of *A. bilimbi* fruit filtrate used as the solvent during the present study. The too low pH may weaken the proton-transfer pathway through ionization suppression by solution H^+^. The low pH may also weaken the electron-transfer pathway by withdrawing the inductive effect (-I) from protonated N-atom, which eventually reduced the phenolic compounds and antioxidant activity of the herbs [28]. Regardless of the acidification effect, the herbs, especially turmeric had a strong antioxidant activity as indicated by the value of IC_50_ ranging from 50 to100 ppm [29].

Throughout the experimental period, the chickens in TRMC and CONT groups gained more weight than those in BLPR and TRPR groups. This was actually not expected as dietary supplementation of acidified herbs was subjected to promote the growth performance of broilers. The exact rationale for the failure of acidified herbs in promoting the growth performance of broiler chicken was not known. The acidic stress due to the dietary supplementation of acidified herbs seemed to be responsible for the lacking effect of acidified herbs on broiler chicken growth. Indeed, over-acidification due to organic acids may compromise nutrient digestibility and alter physiological conditions leading to retarded growth rate of broilers [30]. With regard to citric acid (the most dominant acid in *A. bilimbi* fruit filtrate [26]), Nourmohammadi and Khosravinia [30] documented that while dietary supplementation of 30 g/kg citric acid improved growth performance, the citric acid supplementation at the level of 60 g/kg retarded growth rate of broilers. Islam [31] further confirmed that citric acid should be optimally used in diets to improve the growth performance of broiler chicken at 0.5% and 0.75% for mash and pelleted diets, respectively. Compared to other treated broilers, the broiler chickens supplemented with acidified turmeric had lower FCR values. This may suggest that over-acidification did not occur in the gastrointestinal tract of broilers supplemented with acidified turmeric. In this study, the pH value of acidified turmeric was higher (3.70) than that of acidified black pepper (3.00). In this respect, acidification seemed to be more pronounced, and thus negatively affected the FCR, in the BLPR and TRPR compared to TRMC broilers. In agreement with our suggestion, Khooshechin et al. [32] showed that at higher levels (2 mg/kg) organic acids negatively influenced the FCR of broiler, when compared with that of lower level (1 mg/kg). Aside from the acid effect, the administration of black pepper at 1% of diets seemed to be excessive as Ndelekwute et al. [16] noticed that the dietary supplementation of black pepper at 0.75 and 1% resulted in lower final body weight and higher FCR values, when compared with those at 0, 0.25 and 0.5% of broiler chicken diets. The latter investigators suggested that the adverse impact of black pepper at higher doses may be attributed to the presences of heavy metals (e.g., lead, cadmium and silver), anti-nutritional components and terpernoids, which could negatively affect nutrient utilization and metabolism resulting in poor growth performance of broilers.

Dietary supplementation of acidified black pepper was associated with the increased relative weight of gizzard. This finding was in line with Nourmohammadi and Khosravinia [30] showing an increased gizzard relative weight with dietary citric acid to broilers. Over-acidification, as suspected in BLPR group, may consequently lower nutrient digestibility in broiler chickens [30]. Hence, the increased size of gizzard could be an adaptive response of broiler chickens to the compromised nutrient digestibility. Svihus [33] reported that the increased size of gizzard was the attempt of broilers to increase nutrient digestibility through extended retention time and grinding and mixing of the feeds with digestive enzymes. With regard to effect of black pepper, this herb seemed not to affect the relative weight of gizzard as reported by Al-Kassie et al. [34] and El Tazi et al. [35] who did not observe any impact of black pepper powder (up to 1% from diets) on the relative weight of gizzard. Interesting finding was observed in this study, at which the relative weight of pancreas was lower in BLPR than in other broiler chickens. The reduction in size of pancreas may be associated with the reduced production of pancreatic enzymes [36], which consequently compromised nutrient digestibility of broiler chickens. This may therefore partly explain the lower feed efficiency in BLPR and TRPR birds. Yet, this inference should be taken with caution as we did not measure the nutrient digestibility of broiler chickens in the present study. Black pepper seemed not to affect the size of pancreas in this study as Al-Harthi [37] did not find any effect of black pepper on the pancreas weight of broilers. With regard to the acid effect, the over-acidification has been attributed to the poor nutrient digestibility [30], which may be related to the decreased pancreatic enzymes production due to the decreased size of pancreas. However, published study regarding the effect of acidic stress on the size of pancreas of broilers has not been found to date.

Dietary supplementation of acidified herbs was associated with the reduction in abdominal fat content of broilers. In conjunction with the abdominal fat-lowering effect of turmeric [38] and black pepper [16,35], the organic acid contained in the acidified herbs seemed also to contribute to the lowered abdominal fat deposition of broilers. Sabour et al. [39] showed that dietary administration of organic acids (mixture of citric, lactic, acetic, formic, propionic, phosphoric and butyric acids) lowered abdominal fat content of broilers. They further suggested that feed acidification inhibited glycolysis and stimulated glycogenesis, which eventually decreased abdominal fat deposition.

Dietary supplementation of acidified black pepper powder resulted in greater drumstick as compared to control chickens. Owing to the acid effect, Hossain and Nargis [40] documented that organic acid supplementation increased the relative weight of thigh and drumstick of broiler chickens. They suggested that organic acids improve proteolysis in proventriculus resulting in increased musculature/protein accretion rather than fat accretion (indicated by the lower abdominal fat pad). With respect to the effect of black pepper, the present result was in agreement with El Tazi et al. [35] showing an increased drumstick proportion with supplementing black pepper in broiler chicken diets.

In both breast and thigh meats, the L* values were lower in broiler chickens supplemented with acidified herbs. This result may indicate that dietary supplementation of acidified herbs improved the quality of broiler chicken meats. Karunanayaka et al. [41] suggested that lighter or paler colour of meats was associated with lower pH values, higher drip loss, lower water-holding capacity (WHC) and thus pale-soft-exudative (PSE) meats, which is indicator for quality defects in broiler chicken meats. In this study, dietary supplementation of acidified herbs resulted in increased b* values in both breast and thigh meats. This finding was different from Kralik et al. [42] showing a positive correlation between L* and b* values in breast meats. However, such correlation was not consistent as Karunanayaka et al. [41] and Sugiharto et al. [24] did not find any correlation between L* and b* values in their studies. In the previous study, Sugiharto et al. [24] reported that dietary supplementation of butyric acid and combination of butyric and formic acids decreased the L* and increased the a* and b* values of breast broiler chicken meats. They suggested that organic acids may be essential in preventing the destruction of myoglobin in the muscle tissues, and therefore reducing the paleness and increasing the redness of meats. In this study, although statistically not different, the a* values were higher in the meats of acidified herbs-treated than that in control broiler chickens. In addition, the acid effect of acidified herbs seemed to improve protein digestion in proventriculus resulting in increased muscle protein deposition in broiler chicken meats [40]. The latter condition may consequently increase the redness and reduce the lightness of broiler chicken meats. With regard to the effect of herbs, Łukasiewicz et al. [43] supplemented 0.75% of turmeric powder into diets and found no effect on L*, a* and b* values of broiler chicken meats. Also, Tashla et al. [44] did not find any effect on the L*, a* and b* values of meats when supplementing broiler chicken diets with 1% of black pepper. The absorption and deposition of pigments (especially yellow pigment) in the cutaneous tissue have commonly occurred when the chicks are provided with turmeric powder. Yet, the subcutaneous fat seems to be the most dominant target tissue for pigment deposition, instead of muscle tissues [45] that we investigated in the current study.

## 5. Conclusion

Acidification with *A. bilimbi* fruit filtrate decreased pH values and increased total acids of turmeric and black pepper powders. Dietary supplementation of acidified turmeric reduced abdominal fat deposition and improved meat quality of broiler chickens.
